# Caregivers’ experiences of contributing to patients’ self‐care in Chronic Obstructive Pulmonary Disease: A thematic synthesis of qualitative studies

**DOI:** 10.1111/jan.14942

**Published:** 2021-07-10

**Authors:** Maria Matarese, Roberta Pendoni, Michela Piredda, Maria Grazia De Marinis

**Affiliations:** ^1^ Research Unit of Nursing Sciences Campus Bio‐Medico University of Rome Rome Italy; ^2^ Department of Biomedicine and Prevention University of Rome Tor Vergata Rome Italy

**Keywords:** caregivers, Chronic Obstructive Pulmonary Disease, contributions, literature review, nurses, qualitative studies, self‐care, thematic synthesis

## Abstract

**Aim:**

To identify, analyze and synthesize qualitative studies on caregivers’ experiences of contributions to the self‐care of patients with Chronic Obstructive Pulmonary Disease (COPD).

**Background:**

COPD patients perform daily self‐care behaviours to manage the disease. With aging and disease progression, patients need to rely on the contributions of informal caregivers, usually family members, for disease management. Caregivers’ normal or habitual contributions to patients’ self‐care have not yet been completely investigated.

**Design:**

Thematic synthesis of qualitative studies.

**Data sources:**

CINAHL, EMBASE, PubMed, PsycINFO, Web of Science, Scopus, Emcare and OpenGrey databases were searched from inception to September 2020. The search was restricted to English‐language papers.

**Review methods:**

Studies were critically appraised using the Critical Appraisal Skills Programme checklist. The initial line‐by‐line codes were aggregated into descriptive themes from which analytical themes were derived.

**Results:**

Fifteen papers from nine countries, published 2009–2020, were included. Six analytical themes encompassing 22 descriptive themes were identified and grouped in two overarching themes describing caregivers’ experiences of contributions to patients’ self‐care during the stable and exacerbation phases of COPD. In the stable phases, caregivers contribute through maintaining disease stability, promoting healthy behaviours, fostering normal life and helping perform daily activities. During exacerbations, caregivers contribute through assessing, monitoring and managing symptoms in collaboration with patients or autonomously. They contribute by performing actions, motivating patients, organizing care, sensing variations in symptoms, acquiring knowledge and educating patients, making decisions, communicating and collaborating with healthcare providers and patients and advocating for patients.

**Conclusion:**

This thematic synthesis enlarges knowledge of caregivers’ contributions to patients’ self‐care in COPD, detailing the ways by which caregivers provide care to patients.

**Impact:**

Contributing daily to the self‐care of a family member with COPD is a complex experience. Nurses need to acknowledge the importance of caregivers’ contributions to patient disease management and develop effective educational interventions to support them.

## 1 INTRODUCTION

Chronic Obstructive Pulmonary Disease (COPD) is a progressive lung disease characterized by high mortality, morbidity and economic burden. COPD is the third cause of death worldwide; its estimated global prevalence is 11.7%, and it is expected to increase in the next decades due to population ageing and continued exposure to risk factors. The direct costs of COPD, which include costs of exacerbations, hospitalization, medical visits, medications and rehabilitation, are estimated at $32 billion dollars in U.S. and 38.6 billion euros in Europe (Global Initiative for Chronic Obstructive Lung Disease [GOLD], 2020). The disease course is marked by periods of acute exacerbations of respiratory symptoms (AECOPD), beyond normal day‐to‐day fluctuations, that require a change in regular medication (Rodriguez‐Roisin, 2000). The AECOPD may require the use of home oxygen therapy, increased recourse to medical visits, emergency services and hospitalizations (GOLD, 2020). Patients with COPD perform daily self‐care behaviours directed at preventing, controlling and managing the physical, psychological and social consequences of the disease (Clari et al., 2017). With aging and disease progression, the ability of patients with COPD to perform self‐care decreases, and they need to rely on the contributions of an informal caregiver, usually, a family member or friend, to manage their disease (Nakken et al., 2015). The caregiver contribution (CC) to a patient's self‐care has been defined as ‘the provision of time, effort and support to another person who needs to perform self‐care’ (Vellone et al., 2019, p. 246).

### 1.1 Background

Self‐care of chronic illness, defined as the process of maintaining health through health‐promoting and treatment adherence behaviours (self‐care maintenance), monitoring signs and symptoms of the disease (self‐care monitoring) and managing them when they occur (self‐care management) (Jaarsma et al., 2020), is considered of pivotal importance by healthcare systems worldwide, as it can contribute to reducing the individual burden of the disease, improving quality of life and health outcomes in patients, as well as decreasing the social and economic burden of chronic diseases, reducing, for example, recourse to healthcare services (Allegrante et al., 2019).

While self‐care behaviours of patients with COPD have been widely addressed in studies (Clari et al., 2017; Russel et al., 2018), what caregivers normally do to contribute to patient's self‐care has not yet been studied. Research relating to COPD caregivers has mainly focused on the impact of caregiving on their lives, the various roles fulfilled by caregivers, their information and support needs and the effects of caregiving on patients’ health. Caregivers report negative impacts of caregiving on their physical and psychological health, on their relationship with patients and on financial and employment status (Cruz et al., 2017; Giacomini et al., 2012; Grant et al., 2012). Caregivers feel pressured by the multiple roles (i.e., nurse, physician and psychologist) they need to assume, in addition to being family members, to care for the patients (Giacomini et al., 2012). Such new roles require continuous personal adjustments, and responsibilities increase as the disease progresses (Cruz et al., 2017). Support provided by caregivers has been shown to improve patients’ adherence to medication and smoking cessation (Trivedi et al., 2012), increase participation in pulmonary rehabilitation (Chen et al., 2017) and reduce emergency‐service utilization (Wakabayashi et al., 2011).

All these studies peripherally addressed the specific CCs to patients’ self‐care. A few qualitative studies have been conducted to explore the experiences of caregivers in caring for patients with COPD, and even though they mainly highlight the effect of caregiving on caregivers’ lives, they also provide information about what caregivers do daily for the care of patients. To our knowledge, no qualitative review has summarized the caregivers’ experiences of contributing to patient self‐care in COPD. Knowledge derived from such review might help clinicians understand how caregivers help patients to care for their disease at home, which caregiver practices are helpful, and which ones can be further taught through educational interventions, in order to improve patient health outcomes. Such knowledge can also be beneficial to health policy makers to offer the healthcare, social and financial services needed to support caregivers in accomplishing their role, contributing to reduce the social and economic impact of the disease on healthcare systems.

## 2 THE REVIEW

### 2.1 Aim

The aim of this synthesis of qualitative studies was to systematically identify, analyze and synthesize existing qualitative research on caregivers’ experiences of contributions to the self‐care of people affected by COPD.

### 2.2 Design

The review was conducted according to the thematic synthesis method that allows a new interpretation of a phenomenon (Thomas & Harden, 2008). In fact, through a significant and transparent process, this method helps to go beyond the contents of the original studies by following specific review questions (Thomas & Harden, 2008). A thematic synthesis integrating the results of multiple qualitative studies enlarges the understanding of a phenomenon and provides more useful evidence than a single qualitative study to inform clinical practice and health policy (Barnett‐Page & Thomas, 2009). The review protocol was registered on the Prospective Register of Systematic Reviews (PROSPERO) (reference number CRD42020199676). The review was reported following the Enhancing Transparency in Reporting the Synthesis of Qualitative Research (ENTREQ) guidelines (Tong et al., 2012).

### 2.3 Search method

The CINAHL, EMBASE, PubMed, PsycINFO, Web of Science, Scopus, Emcare, OpenGrey databases and Google Scholar were systematically searched from inception to September 30, 2020. The main search terms used were Chronic Obstructive Pulmonary Disease, self‐care, caregiver and qualitative study, and their synonyms, combined with Boolean operators. The search strategy was adapted to the specific index terms of each database. The search was restricted to articles written in English. A search was also performed on the reference lists of identified papers to retrieve other eligible studies. The search strategy performed on PubMed is reported as an example in Table S1. The PICOS approach was used to define the eligibility criteria (Table 1).

**TABLE 1:** Inclusion/exclusion criteria

Criteria	Description
Population	Informal caregivers aged 18 years and over, including family members, friends and other unpaid individuals who were involved in the care of patients with COPD at any disease stage. Studies conducted on caregivers of chronically ill patients were considered if the experiences of caregivers of COPD patients could be extracted from the results (i.e., findings or quotations of caregivers of COPD patients were reported separately and clearly identified).
Phenomenon of interest	The contributions of informal caregivers to the self‐care of patients with COPD, including any initiative, effort, action, practice or behaviour enacted by caregivers to care for patients in the physical, psychological and social dimensions of well‐being. We included studies where the experiences of caregivers, patients and healthcare providers were reported when the specific contributions related to caregivers could be extracted.
Context	The caregivers’ contributions could take place in any setting, such as home, hospital, nursing home, hospice or long‐term setting.
Types of Studies	Any qualitative studies, including but not limited to phenomenology, ethnography, descriptive, grounded‐theory and mixed‐method designs; for mixed‐method studies, we included only the qualitative components of the studies.

### 2.4 Search outcome

**FIGURE 1:**
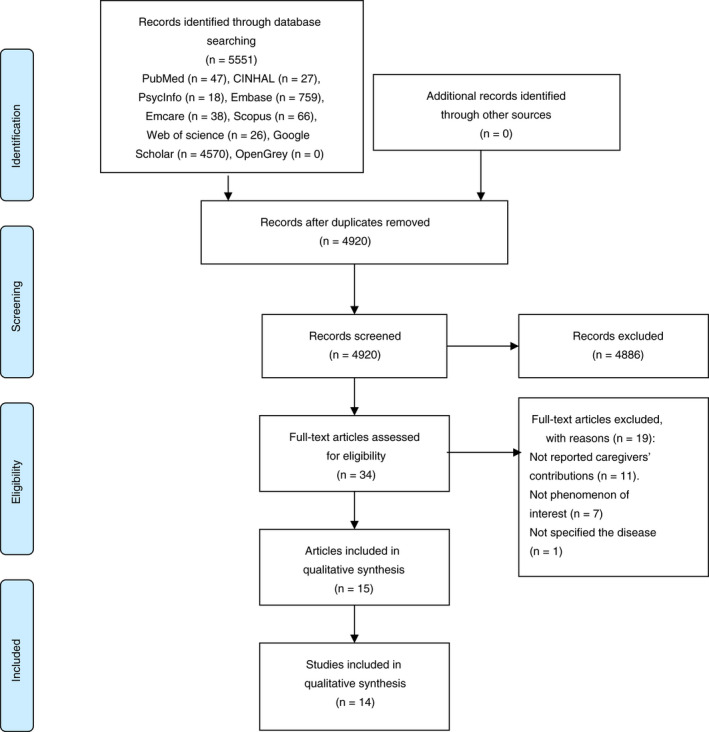
PRISMA flowchart of study selection process

The literature screening was conducted independently by two reviewers (RP, MM), and the results were compared. Any difference was evaluated and resolved when needed with the support of a third reviewer. The electronic searches generated 4926 records after the exclusion of the duplicates; after screening titles and abstracts, 34 articles were eligible and retrieved in full text, and of these, 19 articles were excluded (see Table S2 for the list and reasons for exclusion) (Figure 1). The Zotero software version 5.0 was used to remove duplicates and assist in screening records.

### 2.5 Quality appraisal

Methodological study quality was assessed using the Critical Appraisal Skills Programme (CASP) Checklist for qualitative research (Critical Appraisal Skills Programme, 2018), which considered 10 criteria: clarity and appropriateness of study aims, methodology, design, recruitment strategy, data collection, relationship of researchers with participants, ethics, data analysis, findings and value of research. A response of ‘yes’, ‘no’ or ‘cannot tell’ for each criterion was requested. To be included in the review, studies needed to be allotted ‘yes’ for at least seven criteria. The study quality was evaluated by two reviewers independently and then compared. Any conflicting assessment was resolved by discussion between reviewers or by consulting a third reviewer.

### 2.6 Data extraction and synthesis

A data extraction sheet was used, which included authors, country, study aim, sample characteristics, patient COPD stage, data collection and data analysis method. The thematic synthesis was conducted following a three‐step process: line‐by‐line coding of the primary study's findings, which included the quotes from participants and any other accounts of study author/s about caregiver actions/strategies of contribution to patients included in the ‘results’ or ‘findings’ section of the article as well as in the abstract, tables or discussion section; formulation of descriptive themes from the aggregation of the initial codes according to their conceptual and descriptive similarities; and development of higher‐order analytical themes from the descriptive themes (Thomas & Harden, 2008). The thematic synthesis entails the identification of key concepts from primary studies and their translation into one another, meaning taking concepts from one study and recognizing the same concepts in another study, even though they are expressed using different words (Thomas & Harden, 2008). Data coding was conducted independently by two reviewers (RP, PhD student and MM, expert in qualitative synthesis) and then compared to reach agreement. Preliminary analytical themes were discussed by the reviewers’ team in multiple meetings to reach agreement on the most appropriate thematic structure. The software Atlas.ti version 8 for Windows was used to support the process of data extraction, analysis and synthesis.

## 3 FINDINGS

### 3.1 Study characteristics

The 15 articles described 14 studies. No study was excluded based on the quality appraisal (Table S3). The studies were conducted from 2008 to 2020 in nine countries. They presented the experiences of 163 caregivers, who were aged 20–89 years, mainly females (*n* = 91), spouses/partners (*n* = 69) and caring for patients with severe–very severe COPD (Table 2).

**TABLE 2:** Characteristics of the included qualitative studies in alphabetical order by author(s) (*n* = 15)

Authors, year, country	Aim	Sample	COPD stage	Design	Data collection	Data analysis
Aasbø et al., (2016) Norway	To provide an understanding of how spouses of COPD patients integrate their responsibilities as informal caregivers with their role as spouses and the tensions and challenges involved.	10 spouses F: 6; M: 4 Age range: 60–84 years Retired: 9 Various socioeconomic statuses	Severe‐very severe with comorbidities	Qualitative	Semi‐structured interviews	Thematic analysis
Aasbø et al., (2017) Norway	To investigate how spouses negotiate their role as caregivers with patients and healthcare professionals during acute exacerbations of COPD.	10 spouses F: 6, M: 4 Age range: 61–84 years (7 over 70 years) Various socioeconomic statuses	In oxygen therapy and with comorbidities	Qualitative	Semi‐structured interviews	Thematic analysis
Boyle, (2009) USA	To understand the meaning of the experience of living with a husband with COPD.	10 wives Age range: 57–71 Average married: 41 years Disease length: 3–49 years Socioeconomic characteristics not specified	Not specified	Hermeneutic phenomenology	In‐depth interviews	Interpretative analysis
Bove et al., (2016) Denmark	To explore how spouses of patients with severe chronic COPD experience their role as informal caregiver.	22 spouses F: 13, M: 9 Age range: 61–82 years (mean: 69.4 years) Socioeconomic characteristics not specified	Severe	Qualitative exploratory	Focus groups	Framework method
Essue et al., (2010) Australia	To explore the contribution of caregivers to self‐management partnership with chronically ill care recipients.^a^	7 caregivers Characteristics not specified	Not specified	Qualitative	Semi‐structured interviews	Content analysis
Ferreira et al., (2020) Australia	To understand the experience of living with, and responding to, severe chronic breathlessness in people with COPD from the perspective of the patient and their caregiver.^a^	9 caregivers F: 6, M: 3 Median age: 70 years (IQR: 69–79 years) CO: Oceania: 6; North Eastern Europe: 3 EL: High school: 7, University: 2	Severe	Qualitative	Semi‐structured interview	Constant comparative method
Fotokian et al., (2017) Iran	To illuminate the experiences of empowerment among elderly patients with COPD, their family caregivers and healthcare providers in the Iranian context.^a^	4 family caregivers Characteristics not specified	Not specified	Grounded theory	Semi‐structured in‐depth interviews and field notes	Constant comparative technique
Gysels and Higginson (2009) UK	To investigate the caring experience of caregivers for patients with an advanced progressive illness (lung cancer, COPD, HF) suffering from breathlessness.^a^	2 spouses/partners F: 2, Age: 46, 72 Socioeconomic characteristics not specified	Advanced	Qualitative	Semi‐structured, in‐depth interviews and field notes	Grounded theory approach
Hynes et al., (2012) Ireland	To explore the experiences of informal caregivers providing care in the home to a person with advanced COPD.	11 caregivers F: 9, M: 2 Age range: 20–79 years Spouses. 4, children: 7 Caregiving length: 1–15 years Socioeconomic characteristics not specified	Advanced	Hermeneutic phenomenology	Semi‐structured interviews	Thematic analysis
Philip et al., (2014) Australia	To explore the experiences of current and bereaved informal caregivers of patients with severe COPD.	19 caregivers (9 current, 10 bereaved) F: 10, M: 9 Age range: 28–83 years (mean 70.5 years); Australian: 13. Caregiving length: 4–144 months. spouse/partner: 15, offspring: 3, relative: 1	Severe	Qualitative	Semi‐structured in‐depth interviews	Thematic analysis
Robinson et al., (2018) Australia	To explore the experiences of living with COPD from the perspective of individuals who present frequently to the Emergency Department and their caregivers.^a^	9 caregivers F: 5, M: 4 Socioeconomic characteristics not specified	Attendance of Emergency department ≥3	Descriptive qualitative (mixed‐method study)	Semi‐structured interviews	Thematic analysis
Schafheutle et al., (2018) UK	To describe the activities/strategies patients with COPD utilize after leaving hospital to take their medicines and identify the social network members involved in these activities/strategies.^a^	4 caregivers F: 3, M: 1 Spouses: 2; daughter; 1; family member: 1. Socioeconomic characteristics not specified	From new diagnosis to frequent hospitalizations	Qualitative	Semi‐structured interviews	Thematic analysis
Simpson et al., (2010) Canada	To understand the extent and nature of the ‘burden’ experienced by informal caregivers in advanced COPD.	14 caregivers F: 11, M: 3 Age range: 46–89 years Children: 2, Partner/spouses: 12 Socioeconomic status: mainly low‐middle. EL: Some high school: 6; high school: 4, college: 4	Advanced	Interpretative qualitative	Semi‐structured interview	Thematic network
Spence et al., (2008) Ireland	To explore the needs and experience of family members caring for a person with advanced COPD in the home.	7 caregivers F: 6, M: 1 Age range: 30–65 years. Partners/spouses: 4, children: 2, sibling: 1. Caregiving length: 1 to‐over 4 years. Socioeconomic characteristics not specified	Advanced	Descriptive qualitative	Semi‐structured interviews	Content analysis
Strang et al., (2018) Sweden	To explore the impact of severe COPD on family members’ everyday lives regarding caregiving for a patient in advanced disease, and how relationship between patient and family caregiver is affected.	35 family members F: 10, M: 25 Socioeconomic characteristics not specified	Severe	Qualitative	Focus groups and semi‐structured interviews	Content analysis

Abbreviations: CO, country of origin; COPD, Chronic Obstructive Pulmonary Disease; EL, educational level; F, female; HF, heart failure; M, male.

^a^ Only the experiences of caregivers of patients with COPD were included.

### 3.2 Thematic synthesis findings

Six analytical themes were generated from twenty‐two descriptive themes, derived from 131 codes; the analytical themes were grouped in two overarching themes: Caregiver contributions to patient's self‐care during the stable phase of COPD; and Caregiver contributions to patient's self‐care during AECOPD. The study contributions to the thematic synthesis are reported in Table 3.

**TABLE 3:** Descriptive themes derived from the included studies in alphabetical order (*n* = 22)

Descriptive themes	Aasbø et al., (2016)	Aasbø et al., (2017)	Boyle (2009)	Bove et al., (2016)	Essue et al., (2010)	Ferreira et al., (2020)	Fotokian et al., (2017)	Gysels and Higginson (2009)	Hynes et al., (2012)	Philip et al., (2014)	Robinson et al., (2018)	Schafheutle et al., (2018)	Simpson et al., (2010)	Spence et al., (2008)	Strang et al., (2018)	Total
Acquiring knowledge of COPD			√	√	√		√			√						5
Acquiring knowledge of exacerbation management		√									√					2
Advocating for the patient during exacerbation		√		√	√				√	√						5
Assessing effects of medications during exacerbations		√										√	√			3
Assessing patient's emotional manifestations of exacerbation		√									√			√		3
Assessing patient's physical symptoms of exacerbation	√	√	√	√	√				√			√	√	√	√	10
Collaborating with healthcare personnel	√				√		√			√				√	√	6
Contacting healthcare personnel during exacerbation		√	√	√		√				√	√			√	√	8
Helping patient in ADL	√				√		√			√			√	√	√	7
Helping patient in IADL					√	√	√			√				√	√	6
Maintaining patient's healthy nutrition	√				√								√			3
Maintaining patient's normal habits	√															1
Managing medical equipment during exacerbation	√		√		√					√						4
Managing patient's emotional distress during exacerbation		√				√					√	√				4
Managing patient's medications during exacerbation		√	√								√	√				4
Managing patients’ physical manifestations of exacerbations			√	√		√			√	√	√	√				7
Preserving patient's independence	√															1
Preventing patient's breathlessness	√							√								2
Preventing patient's emotional distress	√			√		√	√							√		5
Preventing patient's risky behaviours	√			√	√			√								4
Promoting patient's adherence to medicine regime	√			√	√							√	√	√		6
Regulating patient's physical activity	√		√	√	√	√								√		6
Total per article	12	8	7	9	11	6	5	2	3	8	6	6	5	9	5	

Abbreviations: ADL, activities of daily living; IADL, instrumental activities of daily living.

#### 3.2.1 Caregivers’ contributions to patients’ self‐care during the stable phase of COPD

This overarching theme includes four analytical themes, derived from twelve descriptive themes (Table 4).

**TABLE 4:** Caregivers’ contributions to patients’ self‐care in the stable phase of COPD

Analytical themes (*n* = 4)	Descriptive themes (*n* = 12)	Codes (*n* = 78)
Theme 1. Contributions in keeping the COPD stable	Preventing patient's breathlessness	Maintaining home free of dust (1) Preventing patient's breathlessness during daily activities (1) Teaching patient breathing technique (8)
	Preventing patient's emotional distress	Avoiding make patient feel a burden (1) (4) Avoiding patient's emotional distress (1) Bringing meaning and joy to patient's life (6) Enhancing the patient's psychosocial capacity (7) Improving patient's morale (7) Keeping patient's spirit up (1) Protecting patient against sadness (4) (7) Providing emotional support (14)
	Promoting patient's adherence to medicine regime	Administering medications (4) (12) Ensuring patient's medication supply (1) Collecting medicines from pharmacy (12) Ensuring adherence to medication regimes (5) (12) Identifying dispensing errors (12) Managing medications (5) (4) (14) (15) Monitoring adherence to treatment regime (13) Motivating patient to follow treatment plans (4) Organizing administration of medicines (12) Reminding patient to take medications (12)
	Collaborating with healthcare personnel	Contacting healthcare services (15) Cooperating with healthcare professionals (7) Encouraging patient to see physician (5) Keeping patient's medical history (10) Liaising with healthcare professional (14) Participating in patient's empowerment (7) Providing help to home care personnel (1)
	Acquiring knowledge of COPD	Acquiring information from medical staff (10) Acquiring information about healthcare and social support benefits (5) Acquiring knowledge of patient care (7) Acquiring knowledge of COPD (4) Acquiring knowledge of patient medications (4) Acquiring skills in patient care (7) Knowing the disease manifestations (3)
Theme 2. Contributions in promoting patient's healthy behaviours	Maintaining patient's healthy nutrition	Attending to daily nutrition (13) Encouraging patient to adhere to diet (5) Monitoring hydration (1) Preparing healthy and easy‐to‐eat food (1) Facilitating ingestion of food when difficult (1)
	Preventing patient's risky behaviours	Avoiding potential risks or dangerous situations (1) Encouraging patient to avoid risky behaviours (5) Negotiating with patient to stop smoking (8) Preventing potential triggers of deterioration (4)
	Regulating patient's physical activity	Arranging daily activities based on the patient's condition (3) Assessing patient's exercise tolerance (3) Performing activities at patient's pace (14) Encouraging patient to take exercise (5) Encouraging patient to increase activity (5) Limiting patient's activities when needed (3) Motivating patient to do physical activities (4) Participating in breathlessness management (6) Preventing overexertion causing breathlessness (6) Regulating patient's activity (3) Slowing down to meet the patient's level of functioning (6) Replacing patient in tasks involving movement (1)
Theme 3. Contributions in fostering patient's normal life	Maintaining patient's normal habits	Supporting the patient's previous habits (1) Maintaining normal life (1) Facilitating patient's participation in everyday routine (1)
	Preserving patient's independence	Maintaining patient's independence and integrity (1)
Theme 4. Contributions in performing ADLs	Helping patient in ADL	Helping in getting out of the bed (10) Helping in bathing (15) Helping in dressing (13) (14) Helping in feeding (14) Helping in personal hygiene (13) (15) Helping in toileting (1) (10) (13) Providing daily physical assistance (5) (10) Providing direct care (14) Providing physical support (7)
	Helping patient in IADL	Assisting in or managing cleaning (5) (6) (15) Assisting in or managing cooking (14) (15) Assisting in or managing finances (5) Assisting in or managing grocery shopping (5) (6) Contacting transportation services (15) Driving to appointments (10) Managing financial resources (14) Managing transportation (5) Providing financial support (7)

Note

Legend: (1) Aasbø et al., 2016; (2) Aasbø et al., 2017; (3) Boyle, 2009; (4) Bove et al., 2016; (5) Essue et al., 2010; (6) Ferreira et al., 2020; (7) Fotokian et al., 2017; (8) Gysels & Higginson, 2009; (9) Hynes et al., 2012; (10) Philip et al., 2014; (11) Robinson et al., 2018; (12) Schafheutle et al., 2018; (13) Simpson et al., 2010; (14) Spence et al., 2008; (15) Strang et al., 2018.

Abbreviations: ADL, activities of daily living; COPD, Chronic Obstructive Pulmonary Disease; IADL, instrumental activities of daily living.

Theme 1 To maintain disease stability, the efforts of the caregivers are directed towards preventing the occurrence of breathlessness and emotional distress in patients, favouring the adherence to prescribed treatments, collaborating with healthcare personnel and acquiring knowledge of COPD. Caregivers prevent patients’ breathlessness by maintaining homes free of dust, teaching breathing techniques and helping them in the daily activities that can provoke dyspnoea. For example, a wife explains:

It really affected his breathing [to go to the toilet]. So I was present much of the time. He didn’t get much time to be alone. I opened the window and gave him the inhaler, and I made sure he was sitting for a while afterwards. (Aasbø et al., 2016, p. 791)

Caregivers try to prevent patients’ emotional distress by avoiding making patients feel a burden, controlling the emotions that trigger patients’ distress (Aasbø et al., 2016) and bringing meaning and joy to patients’ lives, as described by a husband:

I buy her some flowers every fortnight and I put them in there so she can sit at the table and look at them. (…) Just to introduce a bit of color and a variety into the family. I am trying to make it fairly compatible being at home (and I think for the last year she has been home now) I think she said she was happy I think, yeah. (Ferreira et al., 2020, p. 8)

Caregivers consider their manifold contributions important for patients’ adherence to their medical regime: from collecting medicines from the pharmacy to checking patients’ medication intake. Medications management is often complex, as many prescribed medicines have different modes of administration (Bove et al., 2016). A crucial moment is hospital discharge, when medications are usually modified; here, the CC consists of personally administering the medicines to guarantee their correct intake and patient adherence to medication. Caregivers remind family members when to take the medicines and even wake them up at the established time (Schafheutle et al., 2018). To prevent dispensing errors, they prefer to prepare the medicine themselves, for example, asking the pharmacy not to assemble the dosages, even though this can increase the time required for medicine administration (Schafheutle et al., 2018). They motivate patients to take medications using several strategies, even though it is not always an easy task:

When to use a stick and when to use a carrot? The balance is extremely difficult, but you know very well it is a vicious cycle—the less you do, the worse it gets. (Bove et al., 2016, p. 489)

Caregivers collaborate with healthcare professionals in keeping patients’ medical history (Philip et al., 2014), contacting healthcare services on behalf of patients (Strang et al., 2018) or helping to empower patients (Fotokian et al., 2017). Sometimes, caregivers must assume a directive role to force patients to see physicians when they do not want to (Essue et al., 2010). Caregivers recognize the importance of having adequate knowledge of the disease, its treatment and prognosis and the social benefits and healthcare services available in order to care for patients in the best way (Essue et al., 2010):

What would have been helpful and comforting for me [is] to have been fully informed. [The patient] didn’t always want to think about it, but I needed to so I could work out what to do. (Philip et al., 2014, p. 424)

The information is sought from medical staff (Fotokian et al., 2017; Philip et al., 2014) but also acquired through self‐study, personal experience or even collected by chance (Fotokian et al., 2017). Over time, they can become disease experts:

When I saw my mother’s disease progress day by day, I followed up with the doctor. I know a lot about my mother’s disease, about drugs, respiratory aid devices. As the saying goes, I am an ‘expert’. (Fotokian et al., 2017, p. 4)

Theme 2 Caregivers contribute to the promotion of patients’ healthy behaviours, preventing risky behaviours and regulating physical activity. Caregivers prepare nutritive and easy‐to‐eat food to respond to patients’ nutritional needs especially when their appetite is poor (Aasbø et al., 2016). They encourage patients to avoid potentially dangerous behaviours and are continuously alert to avoid situations that can trigger disease worsening (Bove et al., 2016). The most difficult habit to modify in COPD patients is tobacco smoking, and sometimes, caregivers must negotiate with the patient to reduce smoking (Gysels & Higginson, 2009). To prevent the occurrence of dyspnoea, caregivers regulate patients’ physical activity, either setting limits or encouraging them to do more, depending on the patients’ clinical condition (Boyle, 2009). Caregivers learn how to meet the patients’ level of activity tolerance:

Just be patient and let them take their time. When we go to the shops, I don’t rush mum and we just go slow. Be patient. (Ferreira et al., 2020, p. 6)

Theme 3 Caregivers seek to guarantee a normal life for patients, maintaining their usual habits and daily routines and accepting that they may require more time to accomplish them (Aasbø et al., 2016). Maintaining the integrity and independence of their family members is another way of giving them a normal life. One study reports how the wife helped:

(…) For instance, he often managed to make himself a simple breakfast but only if she [the wife] had facilitated it: “I make sure that the spread he uses on his sandwich is easily accessible in the fridge. Not too far down because bending down takes eleven times more energy than not having to. Everything is placed so that he can just take it without having to bend down. So I put the bread on the top shelf in the door of the fridge (…).” (Aasbø et al., 2016, p. 790)

However, it is not always easy for caregivers to understand how much to expect from the patients and how much to take on themselves in everyday situations (Aasbø et al., 2016).

Theme 4 Based on the patients’ level of dependence and disease severity, the caregivers support or replace patients in performing basic activities of daily living (ADLs), such as bathing, personal hygiene, dressing, feeding, toileting and getting out of bed. When the patient is still sufficiently independent, the caregiver is mainly responsible for the most physically demanding instrumental ADLs, such as grocery shopping, house cleaning (Ferreira et al., 2020) or cooking (Spence et al., 2008; Strang et al., 2018). They accompany patients to appointments either by taking them personally or by contacting transport personnel. Caregivers also help patients to manage their finances (Spence et al., 2008) and support them economically when needed (Fotokian et al., 2017).

#### 3.2.2 Caregivers’ contributions to patients’ self‐care during acute exacerbations

Two analytical themes derived from 10 descriptive themes illustrate this overarching theme (Table 5).

**TABLE 5:** Caregivers’ contributions to patients’ self‐care during acute exacerbations of COPD

Analytical themes (*n* = 2)	Descriptive themes (*n* = 10)	Codes (*n* = 53)
Theme 5. Contributions in assessing manifestations of patient's acute exacerbation	Assessing patient's emotional manifestations of exacerbation	Checking for emotional distress (2) Observing psychological manifestations (14) Recognizing the occurrence of the cycle of dyspnoea‐anxiety‐panic attack (11)
	Assessing patient's physical symptoms of exacerbation	Assessing patient's’ symptoms (2) (3) (9) (14) Being alert to identify changes in patient's condition during the night (5) Being aware of signs of deterioration (4) Checking patient's breath during the night (1) Checking patient's body temperature (1) Checking patient's changing needs (5) Interpreting changes in disease conditions (2) Judging patient's conditions (14) Monitoring manifestations of low blood oxygen (2) Monitoring patient by using communication devices at night (3) Monitoring patient's breathing (1) (15) Monitoring possible worsening of symptoms (3) (12) Negotiating symptom interpretation with patient (2) Observing patient during exacerbation (2) Recognizing the manifestations of exacerbation (3) (12)
	Assessing effects of medications during exacerbation	Monitoring medications during exacerbation (2) Monitoring oxygen therapy during the night (13) Assessing side‐effects of medications (12)
Theme 6. Contributions in managing the patient's acute exacerbation	Managing patient's physical manifestations of exacerbation	Decision‐making during symptom exacerbation (9) Developing a plan for emergency (10) Managing acute life‐threating situations (4) (6) Managing exacerbation at the first symptoms (12) Supporting patient in managing exacerbation (3) (11)
	Managing patient's emotional distress during exacerbation	Keeping patient calm during breathlessness (6) (12) Managing patient's anxiety during exacerbation (2) (11)
	Managing medical equipment during exacerbation	Managing patient's oxygen mask during the night (1) Managing medical equipment (oxygen concentrator) (5) Managing technologies (3) Setting up backup systems in case of electricity outage (1) Using medical equipment (10)
	Managing patient's medications during exacerbation	Administering medications during exacerbation (2) (11) (12) Deciding on treatments during exacerbation (3) Regulating medications during exacerbation (2) Managing oxygen during exacerbation (2)
	Advocating for the patient during exacerbation	Advising health professional about the severity of exacerbation (2) Advocating for the patient (5) (9) (10) Being watchdog (5) Claiming involvement in decision‐making with physician (4) (5) Interpreting health information for the patient (5) Using authority to obtain attention from health professional during exacerbation (2)
	Contacting healthcare personnel during exacerbation	Calling emergency services during exacerbation (2) (3) (6) (15) Calling physician during exacerbation (11) Deciding for professional intervention (2) (4) (14) Deciding to go to hospital for treating exacerbation (10) (11) Negotiating medical assistance for the patient during exacerbation (2) Negotiating the need for hospitalization during exacerbation (2) Negotiating with patient about seeking professional help during exacerbation (2)
	Acquiring knowledge of exacerbation management	Knowing the strategy of exacerbation management (11) Learning patient's specific clinical manifestations of exacerbation (2) Learning manifestations of exacerbation from experience (2)

Note

Legend: (1) Aasbø et al., (2016); (2) Aasbø et al., (2017); (3) Boyle (2009); (4) Bove et al., (2016); (5) Essue et al., (2010); (6) Ferreira et al., (2020); (7) Fotokian et al., (2017); (8) Gysels and Higginson (2009); (9) Hynes et al., (2012); (10) Philip et al., (2014); (11) Robinson et al., (2018); (12) Schafheutle et al., (2018); (13) Simpson et al., (2010); (14) Spence et al., (2008); (15) Strang et al., (2018).

Abbreviations: COPD, Chronic Obstructive Pulmonary Disease.

Theme 5 Caregivers are involved in the assessment of emotional and physical manifestations of exacerbations and in monitoring the side‐effects of medicines. Caregivers are aware that breathless patients can suffer from anxiety (Aasbø et al., 2017; Spence et al., 2008) that, in turn, can worsen the dyspnoea; for this reason, they try to observe its inception so as to interrupt the vicious cycle of dyspnoea–anxiety–panic attack (Robinson et al., 2018). After experiencing several exacerbations, caregivers have learnt to recognize and interpret their manifestations (Boyle, 2009; Hynes et al., 2012), such as changes in oxygen saturation (Aasbø et al., 2017), variations in body temperature and breath sounds (Aasbø et al., 2016). However, assessment can be very challenging due to co‐morbidities, ambiguous symptoms and occurrence of new symptoms:

Goodness me, how insecure you can feel when you [don’t] understand. (…) I have learnt that when he starts behaving like a drunkard, to put it that way, then it’s a sign of low oxygen saturation because he becomes all giggly. (Aasbø et al., 2017, p. 614‐615)

Symptoms assessment can be sporadic or constant (Boyle, 2009) and can also occur during the night. Caregivers remain alert even when patients are sleeping (Aasbø et al., 2016; Boyle, 2009; Essue et al., 2010); they can use devices to support them, such as intercoms or portable phones (Boyle, 2009). Patient monitoring is useful to prevent the occurrence of exacerbations:

What I have realised over the last 4 or 5 months is that if you do keep an eye on her and you monitor Mum you can nip [an exacerbation] in the bud. If you look and you see… you recognise the signs. (Schafheutle et al., 2018, p. 1024)

Assessing the side‐effects of medications and the correct administration of oxygen therapy is also considered important by caregivers during exacerbation episodes (Aasbø et al., 2017; Schafheutle et al., 2018), even during the night (Simpson et al., 2010).

Theme 6 Caregivers treat the emotional and physical symptoms of exacerbations, manage patients’ medications and medical equipment, advocate for patients and contact healthcare personnel when needed. To do all that, they need to acquire as much knowledge as possible about management of exacerbations. Caregivers support patients in the actions needed to manage exacerbations (Boyle, 2009; Robinson et al., 2018). Patients may ask their caregiver's help when they are unable to take action (Ferreira et al., 2020), or they may be unaware of changes, and therefore, caregivers need to act for them (Boyle, 2009). Caregivers make decisions based on their experience, empathy and knowledge (Hynes et al., 2012; Robinson et al., 2018). They have an emergency plan to implement in acute life‐threating situations, including when hospitalization is required (Philip et al., 2014). During exacerbations, caregivers try to reduce patients’ anxiety and panic attacks that can worsen dyspnoea, by reassuring patients and calming them down (Ferreira et al., 2020) and communicating safety and control (Aasbø et al., 2017):

‘I just have to reassure him … just slowly try and get his breath back’. ‘The only thing that I can do is… just try and talk her through it. Sometimes it works and other times it doesn’t (…)’. (Robinson et al., 2018, p. 52)

Caregivers decide when and what medications are needed (Boyle, 2009) and administer them to patients (Aasbø et al., 2017; Robinson et al., 2018; Schafheutle et al., 2018) to reduce the symptoms and avoid taking the patient to the emergency department:

If he’s coughing more and bringing up more sputum, it’s an infection. I just take matters into my own hands and say take these (antibiotics). (Robinson et al., 2018, p. 53)

Caregivers manage the oxygen therapy following the healthcare providers’ indications even though it is not always easy: a husband reports that sometimes he increases the amount of oxygen, even if he knows about raising the risk of CO_2_ poisoning, just to give his wife some relief (Aasbø et al., 2017). Caregivers learn to manage medical equipment, such as oxygen masks (Aasbø et al., 2016), oxygen concentrators, nebulizers (Essue et al., 2010) and electric backup systems (Aasbø et al., 2016). However, managing technologies increases the complexity of exacerbation management (Boyle, 2009). When the patients are unable to participate in exacerbation management, caregivers assume the role of patient's ‘advocate’ (Essue et al., 2010; Hynes et al., 2012; Philip et al., 2014); they make decisions on behalf of the patients and interpret the information, especially when the patients have memory impairment and difficulty in understanding treatments, belong to a different culture or are critically ill (Essue et al., 2010). For caregivers, watchfulness over care and decision‐making are also important during patient hospitalization:

Because I’m the one that’s dealing with it when X comes home…and then every day I go up and I check… I’m just watching everything that’s going on up there… because after all, if I don’t look after X, who will look after X, do you know? (Hynes et al., 2012, p. 1072)

Caregivers use their authority to make professionals take patients’ exacerbations seriously (Aasbø et al., 2017). They believe that they must be listened to and taken into consideration by physicians (Bove et al., 2016) as they manage the sick person for most of the time:

I started to ask him [specialist] questions. He said “Excuse me madam”, and I just said to him “Excuse me, don’t you speak to me like that … he’s my husband, he’s sick, you’re his doctor, now I want to know what’s wrong with him.” Then he backed down. (Essue et al., 2010, p. 417)

When the patient's condition becomes critical, caregivers call the general practitioner for a home visit (Robinson et al., 2018), the emergency services for consultation (Aasbø et al., 2017; Boyle, 2009; Ferreira et al., 2020; Strang et al., 2018) or they take the patient to hospital. Making the decision about who and when to call for help is not always easy (Aasbø et al., 2017). When the healthcare personnel underestimate the severity of the condition, caregivers need to negotiate with them for the assistance needed based on their own judgement and expertise, trying to communicate certainty to professionals (Aasbø et al., 2017). They also negotiate with the patients when seeking for professional help; sometimes, patients want to wait and see if they can manage the exacerbation on their own. This causes a dilemma in caregivers, as delay in seeking help could worsen the patient's condition (Aasbø et al., 2017). After experiencing several exacerbations, caregivers have learnt what kinds of manifestations require attention (Aasbø et al., 2017) and how to manage the patients’ acute events (Robinson et al., 2018).

#### 3.2.3 Modalities of caregivers’ contributions to patients’ self‐care

Based on the study findings, we identified nine modalities through which caregivers contribute to patients’ self‐care:

- *Practical contribution* that entails the observable actions that caregiver performs in collaboration with or in place of the patient, such as administering medications, collecting medicines from the pharmacy or bathing the patient;
- *Motivational contribution* that includes the encouragement provided by caregiver to elicit patient's self‐care behaviours, for example, encouraging patients to perform physical exercise, recommending actions in case of symptoms or persuading them to call physician for check‐ups;
- *Organizational contribution* that encompasses the arrangements and plans made by caregivers to assure patients’ care, including organizing the pick‐up of medicines at the pharmacy, managing the administration of patients’ medicines or arranging patients’ daily activities;
- *Perceptive contribution* that includes the assessment and monitoring of patients’ condition made by using the senses (seeing, hearing, smelling, touching); for example, observing the patient's skin, listening to breath sounds or touching the body to assess temperature;
- *Educational contribution* that includes acquiring information about the disease and learning the skills needed to provide care as well as to educate the patient; for example, learning what the signs of an exacerbation are or teaching patients breathing techniques;
- *Cognitive contribution* that comprises the mental activities needed to interpret and judge the patient's condition and decide on the course of action; for example, recognizing the signs of an exacerbation, making decisions about the treatment during acute events or identifying dispensing errors in medications;
- *Communication contribution* that entails exchanging information with the healthcare personnel and the patient about care; for example, calling the emergency services, contacting healthcare personnel to provide suggestions or advising physicians about patients’ critical conditions;
- *Collaborative contribution* that entails collaborating with healthcare personnel and patient regarding patient care, such as cooperating with healthcare professionals to empower the patient, collaborating with patients in breathlessness management;
- *Advocacy contribution* that comprises the activities performed by caregiver to assure that the patient receives the best care, and his/her voice is heard by healthcare providers, especially when patient is unable to do it.

**FIGURE 2:**
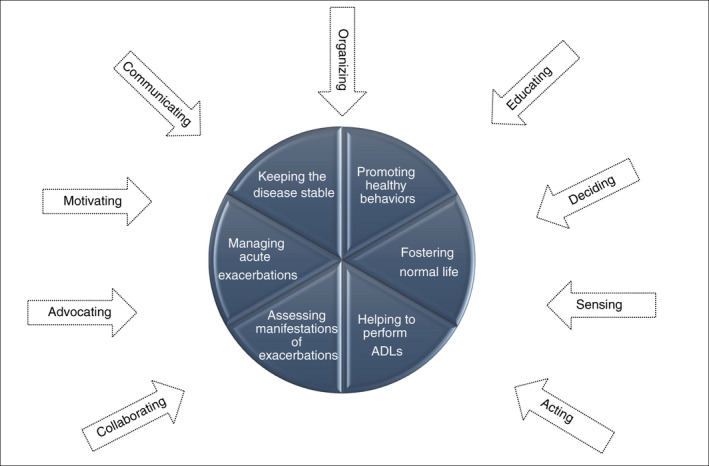
Model of caregivers’ contributions to self‐care of patients with COPD

A model synthesizing the CCs to patients’ self‐care derived from findings is reported in Figure 2.

## 4 DISCUSSION

To the best of our knowledge, this is the first review of qualitative studies to synthesize the experiences of CCs to the self‐care of patients with COPD. Although previous qualitative reviews have described the multiple roles played by caregivers in caring for the patients (Giacomini et al., 2012), they have not identified the specific daily CCs to patient self‐care, highlighting mainly the burden and the increase in responsibility due to caregiving. In our review, we found that, in the stable phase, caregivers contribute with a broad range of practices directed towards preventing patients’ breathlessness and emotional distress, promoting adherence to medicine regimes, establishing collaboration with healthcare providers, acquiring information about COPD, maintaining appropriate nutrition, regulating physical activities, discouraging risky behaviours, maintaining a normal life and helping patients in ADLs. During the AECOPD, caregivers’ contributions are directed towards assessing and monitoring the physical and emotional manifestations of the exacerbation as well as the side effects of medications and managing such manifestations by handling medical equipment and medications, advocating for the patients to assure the best care, contacting healthcare services for help and acquiring information to deal correctly with the exacerbations. Previous research showed that CCs can be effective in promoting smoking cessation in COPD patients (Trivedi et al., 2012), adherence to medicine prescriptions (Ladner et al., 2020; Trivedi et al., 2012), increasing exercise capacity and reducing emergency visits when caregivers cohabit with patients (Wakabayashi et al., 2011).

Our review shows that caregivers can use several modalities to contribute to patients’ self‐care, including performing practical activities, motivating patients, organizing care, sensing variations in symptoms and signs, educating, making decisions, communicating and collaborating with healthcare services and patients and advocating for the patients. Even though previous studies identified a few CCs to patients self‐care, such as providing information and direct care, encouraging treatment adherence, acting as an advocate, offering emotional and psychosocial support (Bryant et al., 2016), delivering practical support in housework, garden work, shopping and transportation (Gautun et al., 2012), we found a broader range of contributions that can be explained by the scarce exploration of this phenomenon in COPD and by the richness of information derived from the analyses of several qualitative studies.

Our results suggest that caregivers contribute to patient's self‐care in three dimensions of self‐care, as posited in the specific‐situation theory of Caregiver Contribution to Self‐care of Patients with Heart Failure (HF) (Vellone et al., 2019): contribution to patient self‐care maintenance, in which caregivers help to maintain the stability of patients’ disease and their physical and psychological well‐being; contribution to symptom perception and monitoring; and contribution to self‐care management, in which they respond to the acute exacerbation phases. There are many similarities between HF and COPD: both are long‐term conditions that affect older patients with comorbidities and are characterized by dyspnoea, fatigue, reduced ability to exercise and recurrent exacerbations (Noonan et al., 2019), and these resemblances lead to very similar CCs to self‐care (Buck et al., 2015).

The trajectory of COPD does not follow a continuous descending curve from early to terminal stages as occurs in other chronic diseases, but it shows a roller‐coaster pattern, with ups and downs, due to the AECOPD, that intensify in duration, severity and frequency as the disease progresses (Giacomini et al., 2012). Consequently, the CCs to patient self‐care also follow these ups and downs; caregivers alternate periods in which their contributions increase during the acute exacerbations and periods in which they reduce their contributions, until the patient's health declines to the point of requiring continuous assistance.

Despite many contributions provided by caregivers to patient care, we found that caregivers learn how to support patients by self‐learning, by experience and trial and errors and rarely report being supported in their learning process by healthcare providers. Even when caregivers are included in education programs on patient self‐management, healthcare professionals do not formally address their role (Bryant et al., 2016).

### 4.1 Implications for practice and research

The results of our review help nurses to recognize the broad range of activities that caregivers carry out to take care of COPD patients at home. As most of the care, especially for older and severely ill patients, is performed by caregivers, nurses should consider caregivers as essential allies in patient care and establish sound collaboration for the patient's disease management. Nurses should provide information and disease‐related education in every encounter with the caregivers, for example, during emergency services visits and/or patient hospitalization, before patient discharge, at patient clinic visits and home care; moreover, they should include caregivers in self‐management education programs considering them as target of their interventions together with the patients. Jointly with the caregiver, nurses should screen the CC practices to patient's self‐care and assess which are the most effective and which ones can be modified to better adjust to the needs of patient and caregiver. Nurses should also facilitate the access of caregivers to the social and financial resources that could help them to fulfil their role.

We did not find any qualitative study reporting experiences of CCs during palliative care, although a few of the studies identified considered patients in advanced stages that referred to palliative assistance. Future studies should investigate how caregivers are involved in palliative care and what their contributions are. Additionally, we suggest conducting further qualitative studies exploring experiences of CCs to patient self‐care using different qualitative approaches, such as ethnographic design, that would permit CCs to be observed in the home contexts or longitudinal qualitative research in order to explore the progression of CCs to patient self‐care over time.

### 4.2 Limitations

The review has some limitations. First, the thematic synthesis relies on the interpretations and published quotes of the authors of the primary studies, and it not possible to know how representative these were of the original data. Second, only studies written in English were included in the review, and this could have limited the range of possible experiences of contributions of caregivers from different cultures. Third, due to the limited description of participants’ characteristics provided in several studies, no difference in caregiver experiences based on age, sex, type of caregivers, and sociocultural background could be derived from the findings. Fourth, we identified studies that mainly explored the experiences of caregivers of patients in advanced stages of COPD; therefore, we do not know whether these contributions are applicable to patients in the early stages, and if so, which ones. Finally, as almost all the studies were conducted in Europe, Australia and North America (*n* = 14), the transferability of our findings to other geographical areas could be limited.

## 5 CONCLUSION

This thematic synthesis of qualitative studies expands knowledge about CCs to patients’ self‐care during the stable and AECOPD, deriving these contributions from the direct experiences of caregivers. Caregivers can contribute in several ways to patients’ self‐care, and their contributions are relevant during the whole course of the disease. Caring for a family member with COPD is a complex experience, and healthcare providers, especially nurses, should acknowledge the importance of CCs to the patient's disease management and consider the patient–caregiver dyad as a single unit of care. Nurses need to assess not only the self‐care practices of patients with COPD but also the CCs to those practices in all the healthcare settings where they can meet caregivers—hospitals, clinics, rehabilitation centres, or at home—especially in the advanced stages of the disease, as these contributions are indispensable for disease management. Therefore, nurses need to consider caregivers as essential partners in the process of patient care, and effective educational interventions to increase caregivers’ capacity to support COPD patients’ care should be planned.

## CONFLICT OF INTEREST

The authors have declared no conflict of interest.

## AUTHOR CONTRIBUTIONS

Maria Matarese: review conception and design, literature search, data analysis and synthesis, interpretation of data, draft of manuscript. Roberta Pendoni: literature search, data extraction and data analysis, draft of the manuscript. Maria Grazia De Marinis: critically revision of the manuscript, data synthesis. Michela Piredda: critically revision of the manuscript, data synthesis. All the authors approved the final version of the article and agreed to be accountable for all aspects of the work in ensuring that questions related to the accuracy or integrity of any part of the work are appropriately investigated and resolved.

### PEER REVIEW

The peer review history for this article is available at https://publons.com/publon/10.1111/jan.14942.

## Supporting information

Table S1

Table S2

Table S3
