# Supplementary material for: Caregivers’ experiences of contributing to patients’ self‐care in Chronic Obstructive Pulmonary Disease: A thematic synthesis of qualitative studies
Source: J Adv Nurs. 2021 Jul 10;77(10):4017–34. doi: 10.1111/jan.14942 (PMC8518034; doi:10.1111/jan.14942)
Supplement: Supplementary file 1 — Table S1 [file JAN-77-4017-s003.docx]

**Supplementary file**

**TABLE S1** PubMed search

| N. | Query | Records |
| --- | --- | --- |
| #1 | "pulmonary disease, chronic obstructive"[MeSH Terms] OR "Chronic Obstructive Airway Disease"[Title/Abstract] OR "Chronic Obstructive Lung Disease"[Title/Abstract] OR "chronic obstructive pulmonary disease"[Title/Abstract] OR "COPD"[Title/Abstract] OR "COAD"[Title/Abstract] | 74,384 |
| #2 | "self care"[Title/Abstract] OR "self monitoring"[Title/Abstract] OR "self maintenance"[Title/Abstract] OR "self efficacy"[Title/Abstract] OR "chronic illness management"[Title/Abstract] OR "self management"[Title/Abstract] OR "disease management"[Title/Abstract] OR "self administration"[Title/Abstract] OR "self regulation"[Title/Abstract] OR "self care"[MeSH Terms] | 124,095 |
| #3 | "qualitative stud*"[Title/Abstract] OR "phenomenolog*"[Title/Abstract] OR "qualitative"[Title/Abstract] OR "qualitativ*"[Title/Abstract] OR "Grounded Theory"[Title/Abstract] OR "interview"[Publication Type] OR "interviews as topic"[MeSH Terms] OR "interviews"[Title/Abstract] OR "Semi-Structured Interview"[Title/Abstract] OR "Unstructured Interview"[Title/Abstract] OR "observation*"[Title/Abstract] OR "Thematic Analysis"[Title/Abstract] OR "theme"[Title/Abstract] OR "themed"[Title/Abstract] OR "themes"[Title/Abstract] OR "Audio recording"[Title/Abstract] OR "Audio recording"[Title/Abstract] OR "audio record*"[Title/Abstract] OR "audiotap*"[Title/Abstract] OR "audio tap*"[Title/Abstract] OR "qualitative research"[MeSH Terms] | 1,245,265 |
| #4 | "care partners"[Title/Abstract] OR "carers"[Title/Abstract] OR "family members"[Title/Abstract] OR "relatives"[Title/Abstract] OR "spouses"[Title/Abstract] OR "partners"[Title/Abstract] OR "caregiving"[Title/Abstract] OR "care-givers"[Title/Abstract] OR "children"[Title/Abstract] OR "caregivers"[MeSH Terms] | 1,130,441 |
| #5 | #1 AND #2 AND #3 AND #4 | 47 |

Limits: [Date - Publication]: from inception to 2020/9/30, [language]: English
