# Supplementary material for: Caregivers’ experiences of contributing to patients’ self‐care in Chronic Obstructive Pulmonary Disease: A thematic synthesis of qualitative studies
Source: J Adv Nurs. 2021 Jul 10;77(10):4017–34. doi: 10.1111/jan.14942 (PMC8518034; doi:10.1111/jan.14942)
Supplement: Supplementary file 2 — Table S2 [file JAN-77-4017-s001.docx]

**Supplementary file**

**Table S2 Article excluded after reading full text**

| **Articles** | **reasons for exclusion** |
| --- | --- |
| Andersen, I. C., Thomsen, T. G., Bruun, P., Bødtger, U., & Hounsgaard, L. (2017). Patients' and their family members' experiences of participation in care following an acute exacerbation in chronic obstructive pulmonary disease: A phenomenological‐hermeneutic study. *Journal of Clinical Nursing*, 26(23-24), 4877-4889. | Not reported contributions of caregivers |
| Bergs, D. (2002). "The Hidden Client"-women caring for husbands with COPD: their experience of quality of life. *Journal of Clinical Nursing*, *11*(5), 613-621. | Not reported contributions of caregivers |
| Ek, K., Ternestedt, B. M., Andershed, B., & Sahlberg-Blom, E. (2011). Shifting life rhythms: couples’ stories about living together when one spouse has advanced chronic obstructive pulmonary disease. *Journal of Palliative Care*, 27(3), 189-197. | Not reported contributions of caregivers |
| Farquhar, M., Penfold, C., Benson, J., Lovick, R., Mahadeva, R., Howson, S., ... & Ewing, G. (2017). Six key topics informal carers of patients with breathlessness in advanced disease want to learn about and why: MRC phase I study to inform an educational intervention. *PloS one,* 12(5), e0177081. | No phenomenon of interest |
| Figueiredo, D., Jácome, C., Gabriel, R., & Marques, A. (2016). Family care in chronic obstructive pulmonary disease: what happens when the carer is a man?. *Scandinavian Journal of Caring Sciences,* 30(4), 721-730. | Not reported contributions of caregivers |
| Fusi-Schmidhauser, T., Froggatt, K., & Preston, N. (2020). Living with Advanced Chronic Obstructive Pulmonary Disease: A Qualitative Interview Study with Patients and Informal Carers. COPD: *Journal of Chronic Obstructive Pulmonary Disease*, 17(4):410-418. | Not reported contribution of caregivers |
| Gabriel, R., Figueiredo, D., Jácome, C., Cruz, J., & Marques, A. (2014). Day-to-day living with severe chronic obstructive pulmonary disease: towards a family-based approach to the illness impacts. *Psychology & Health*, 29(8), 967-983. | Not reported contributions of caregivers |
| Gullick, J., & Stainton, M. C. (2008). Living with chronic obstructive pulmonary disease: developing conscious body management in a shrinking life‐world. *Journal of Advanced Nursing*, 64(6), 605-614. | Not reported contributions of caregivers |
| Hasson, F., Spence, A., Waldron, M., Kernohan, G., McLaughlin, D., Watson, B., & Cochrane, B. (2009). Experiences and needs of bereaved carers during palliative and end-of-life care for people with chronic obstructive pulmonary disease. *Journal of Palliative Care,* 25(3), 157-163. | Not reported contributions of caregivers |
| Jonsdottir, H. (2007). Research-as-if-practice: a study of family nursing partnership with couples experiencing severe breathing difficulties. *Journal of Family Nursing*, 13(4), 443-460. | Not specified the disease causing breathing difficulties |
| Kanervisto, M., Kaistila, T., & Paavilainen, E. (2007). Severe chronic obstructive pulmonary disease in a family’s everyday life in Finland: perceptions of people with chronic obstructive pulmonary disease and their spouses. *Nursing & Health sciences,* 9(1), 40-47. | No phenomenon of interest |
| Karasouli, E., Munday, D., Bailey, C., Staniszewska, S., Hewison, A., & Griffiths, F. (2016). Qualitative critical incident study of patients’ experiences leading to emergency hospital admission with advanced respiratory illness. *BMJ open,* 6(2), e009030. | No phenomenon of interest |
| Lindqvist, G., Albin, B., Heikkilä, K., & Hjelm, K. (2013). Conceptions of daily life in women living with a man suffering from chronic obstructive pulmonary disease. *Primary Health Care Research & Development,* 14(1), 40-51. | No phenomenon of interest |
| Lindqvist, G., Heikkilä, K., Albin, B., & Hjelm, K. (2013). Conceptions of daily life in men living with a woman suffering from chronic obstructive pulmonary disease. *Primary Health Care Research & Development,* 14(2), 140-150. | No phenomenon of interest |
| Mirzaei, M., Aspin, C., Essue, B., Jeon, Y. H., Dugdale, P., Usherwood, T., & Leeder, S. (2013). A patient-centred approach to health service delivery: improving health outcomes for people with chronic illness. *BMC Health Services Research,* 13(1), 251. | No phenomenon of interest |
| Schunk, M., Schulze, F., & Bausewein, C. (2019). What constitutes good health care for patients with breathlessness? Perspectives of patients, caregivers, and health care professionals. *Journal of Palliative Medicine*, 22(6), 656-662. | Not reported contribution of caregivers |
| Seamark, D. A., Blake, S. D., Seamark, C. J., & Halpin, D. M. (2004). Living with severe chronic obstructive pulmonary disease (COPD): perceptions of patients and their carers: An interpretative phenomenological analysis. *Palliative medicine*, 18(7), 619-625. | Not reported contribution of caregivers |
| Wang, Y., Haugen, T., Steihaug, S., & Werner, A. (2012). Patients with acute exacerbation of chronic obstructive pulmonary disease feel safe when treated at home: a qualitative study. *BMC Pulmonary Medicine,* 12(1), 45. | Not reported contributions of caregivers |
| Ward, N. J., Jowsey, T., Haora, P. J., Aspin, C., & Yen, L. E. (2011). With good intentions: complexity in unsolicited informal support for Aboriginal and Torres Strait Islander peoples. A qualitative study. *BMC Public Health,* 11(1), 686. | No phenomenon of interest |
