# Supplementary material for: Caregivers’ experiences of contributing to patients’ self‐care in Chronic Obstructive Pulmonary Disease: A thematic synthesis of qualitative studies
Source: J Adv Nurs. 2021 Jul 10;77(10):4017–34. doi: 10.1111/jan.14942 (PMC8518034; doi:10.1111/jan.14942)
Supplement: Supplementary file 3 — Table S3 [file JAN-77-4017-s002.docx]

**Table S3 Quality critical appraisal of the included studies according to Critical Appraisal Skills Programme (CASP) Qualitative Checklist (**[**https://casp-uk.net/wp-content/uploads/2018/01/CASP-Qualitative-Checklist-2018.pdf**](https://casp-uk.net/wp-content/uploads/2018/01/CASP-Qualitative-Checklist-2018.pdf)**)**

|  | Aasbø  et al., 2016 | Aasbø  et al., 2017 | Boyle  et al.  2009 | Bove  et al.  2016 | Essue  et al., 2010 | Ferreira  et al.  2020 | Fotokian  et al., 2017 | Gysels &  Higginson,  2009 | Hynes  et al., 2012 | Philip  et al., 2014 | Robinson  et al., 2018 | Schafheutle  et al.,  2018 | Simpson  et al., 2010 | Spence  et al., 2014 | Strang  et al., 2018 |
| --- | --- | --- | --- | --- | --- | --- | --- | --- | --- | --- | --- | --- | --- | --- | --- |
| 1. Was there a clear statement of the aims of the research? | Y | Y | Y | Y | Y | Y | Y | Y | Y | Y | Y | Y | Y | Y | Y |
| 2. Is a qualitative methodology appropriate? | Y | Y | Y | Y | Y | Y | Y | Y | Y | Y | Y | Y | Y | Y | Y |
| 3 Was the research design appropriate to address the aims of the research? | Y | Y | Y | Y | Y | Y | Y | Y | Y | Y | Y | Y | Y | Y | Y |
| 4 Was the recruitment strategy appropriate to the research aims? | Y | Y | Y | Y | Y | Y | Y | Y | Y | Y | Y | Y | Y | Y | Y |
| 5 Was the data collected in a way that addressed the research issue? | Y | Y | Y | Y | Y | Y | Y | Y | Y | Y | Y | Y | Y | Y | Y |
| 6 Has the relationship between research and participants been adequately considered? | N | N | N | N | N | N | N | N | N | N | N | N | N | N | N |
| 7 Have ethical issues been taken into consideration | Y | Y | Y | Y | CT | Y | Y | Y | Y | Y | Y | Y | Y | Y | Y |
| 8 Was the data analysis sufficiently rigorous? | Y | Y | Y | Y | Y | Y | Y | Y | Y | Y | Y | Y | Y | Y | Y |
| 9 Is there a clear statement of findings? | Y | Y | Y | Y | Y | Y | Y | Y | Y | Y | Y | Y | Y | Y | Y |
| 10. How valuable is the research? | Y | Y | Y | Y | Y | Y | Y | Y | Y | Y | Y | Y | Y | Y | Y |
| Total yes | 9 | 9 | 9 | 9 | 8 | 9 | 9 | 9 | 9 | 9 | 9 | 9 | 9 | 9 | 9 |

Y= Yes; CT =Can’t tell; N = No
